# Psychosocial characteristics pattern correlated with suicidal ideation and non-suicidal self-injury among nurse staff: a latent profile analysis

**DOI:** 10.1186/s12912-024-01970-5

**Published:** 2024-04-26

**Authors:** Xuehua Li, Changmian Ding, Guizhi Li, Zhizhou Duan

**Affiliations:** 1 The Medical Record Management Department, The People’s Hospital of Dehong, Yunnan, China; 2 Preventive health service, Jiangxi provincial people’s Hospital, The First Affiliated Hospital of Nanchang Medical College, Nanchang, Jiangxi China

**Keywords:** Nurse stuff, Suicidal ideation, Non-suicidal self-injury, Psychosocial characteristics, Latent profile analysis

## Abstract

**Background:**

Nurses frequently endure elevated levels of psychosocial stress, which often correlates with an increased suicide risk. This study aimed to investigate the impact of latent psychosocial characteristic patterns on suicidal ideation and non-suicidal self-injury among nursing staff.

**Method:**

Participants were recruited from the Dehong districts of Yunnan province, China, between July 11th and July 26th, 2022. Subgroups were identified using variables linked to suicidal ideation and non-suicidal self-injury, including perceived cognitive deficits, anxiety symptoms, depressive symptoms, resilience, social support, childhood trauma, loneliness, and sleep quality. Measurement tools included the Perceived Deficit Questionnaire-5-item (PDQ-5), Generalized Anxiety Disorder-7 items (GAD-7), Patient Health Questionnaire (PHQ-9), Connor-Davidson Resilience Scale-10 items (CD-RISC10), Multidimensional Scale of Perceived Social Support (MSPSS), Childhood Trauma Questionnaire-Short Form (CTQ-SF), Three-Item Loneliness Scale, and a single-item sleep quality scale.

**Results:**

Latent profile analysis (LPA) revealed four distinct psychosocial characteristic patterns: “class 1,” “class 2,” “class 3,” and “class 4.” Compared to class 2, individuals in class 1 had a sixfold increased risk of suicidal ideation (OR = 6.59, 95%CI = 4.42–9.81) and a fivefold increased risk of non-suicidal self-injury (OR = 5.13, 95%CI = 3.38–7.78). Similarly, class 4 individuals had twice the risk of suicidal ideation (OR = 2.13, 95%CI = 1.25–3.62) and non-suicidal self-injury (OR = 2.13, 95%CI = 1.25–3.65) compared to class 2. Conversely, class 3 individuals had a lower risk of suicidal ideation (OR = 0.21, 95%CI = 0.11–0.42) and non-suicidal self-injury (OR = 0.15, 95%CI = 0.07–0.36) than class 2. Additionally, divorced/other marital status individuals had a higher risk of suicidal ideation (OR = 2.34, 95%CI = 1.02–5.35) and non-suicidal self-injury (OR = 2.58, 95%CI = 1.01–6.65) compared to married individuals, while unmarried individuals had a lower risk of suicidal ideation (OR = 0.58, 95%CI = 0.37–0.91).

**Conclusions:**

The study identified eight important psychosocial factors divided into four latent pattern classes. Individuals in “class 1” and “class 4” were more likely to have a higher risk of suicidal ideation and non-suicidal self-injury, while those in “class 3” were more likely to have a lower risk of both outcomes. It is suggested that further research should focus on “class 1” and “class 4” for targeted intervention.

## Introduction

Suicide presents a significant public health concern globally. According to a 2019 report by the World Health Organization (WHO), approximately 700,000 individuals worldwide die by suicide annually, equating to a suicide rate of 11.4 per 100,000 people [1]. Despite a noticeable decline in suicide rates in China over recent decades [2], specific demographic groups continue to exhibit higher rates of suicide. Nurses, constituting a unique professional cohort characterized by long working hours, complex job responsibilities, and heightened occupational risks, often encounter substantial mental stress. Consequently, the incidence of suicide among nurses is notably higher compared to the general population [3, 4]. However, research focusing on suicide and suicide-related issues specifically among nurses remains limited [5, 6]. This highlights the urgent need for studies that delve deeper into the unique challenges faced by nurses regarding mental health and well-being.

Suicidal ideation encompasses thinking about, contemplating, or making plans for suicide [7]. Non-suicidal self-injury (NSSI) involves deliberate, self-inflicted harm to the body’s surface without the intent to end one’s life [8]. Common methods reported include cutting, scratching, scraping, hitting or banging, and carving [9, 10]. Both suicidal ideation and non-suicidal self-injury can lead to adverse outcomes such as physical injury, hospitalization, loss of personal freedom, and even suicide deaths [7]. Therefore, beyond suicide deaths, attention should also be directed towards addressing suicidal ideation and non-suicidal self-injury.

Previous research has consistently demonstrated the significant association between psychosocial factors and suicidal ideation. For instance, a study involving 2,549 rural empty-nest older adults revealed that individuals with cognitive frailty may have an increased likelihood of experiencing suicidal ideation [11]. Similarly, a study among 11,133 Chinese university students identified anxiety and depressive symptoms as reliable risk factors for suicidal ideation, while social support was recognized as a protective factor against suicidal ideation [12]. Moreover, childhood adversities, particularly emotional abuse, were found to be positively associated with suicidal ideation [13, 14], with low levels of resilience correlated with its occurrence [15], Childhood maltreatment may heighten the risk of suicidal ideation by diminishing resilience levels [14]. Loneliness was also established as a predictor of heightened anxiety symptoms and the occurrence of suicidal ideation [16], while sleep quality was significantly related to suicidal ideation, with insufficient sleep directly increasing the risk of suicidal ideation [17, 18].

Parallel studies have highlighted the crucial role of psychosocial factors in the development of non-suicidal self-injury. In a study of 1,771 Chinese adolescents, it was found that depressive and anxiety symptoms not only directly impacted non-suicidal self-injury and suicidal attempts but also indirectly elevated the risk of these behaviors by exacerbating sleep quality issues [19]. Another study involving 1,610 Chinese adolescents reported that emotional neglect could directly elevate the risk of non-suicidal self-injury and indirectly heighten the risk by increasing the likelihood of developing social anxiety or insomnia [20]. Additionally, resilience and social support act as protective factors for reducing non-suicidal self-injury [13, 21], whereas a higher level of loneliness has been significantly associated with an increased risk of non-suicidal self-injury [22, 23].

The combined effects of multiple psychosocial characteristics on suicidal ideation and non-suicidal self-injury among nursing staff remain unclear, despite exploring individual psychosocial factors. Traditional variable-centered analysis often overlooks the heterogeneity and combined effects of these characteristics. To address this gap, our study employed latent profile analysis (LPA), a person-centered approach aimed at uncovering specific population patterns. In all, the primary objectives of the study were to employ LPA to reveal latent class patterns of psychosocial characteristics and subsequently investigate their association with suicidal ideation and non-suicidal self-injury among nurse staff. In addition, through LPA, we aimed to capture the nuanced interactions and combined effects of various psychosocial factors in relation to these critical mental health outcomes. By investigating the impact of latent psychosocial characteristic patterns on suicidal ideation and non-suicidal self-injury among nursing staff, our study contribute to the development of targeted interventions and support systems for this vulnerable population.

## Method

### Participant and procedure

The data for this study were obtained from a cross-sectional study conducted in the Dehong districts of Yunnan province between July 11th and July 26th, 2022. Situated in the southwestern region of China, Dehong is an ethnic border district with a population of approximately 1.32 million individuals as of the end of 2021. It shares a 503.8-kilometer-long border with Myanmar, extending along its northern, western, and southern perimeters [24]. The proximity to Myanmar and the diverse population in the region present a unique context for examining the psychosocial characteristics and mental health outcomes among nursing staff. The participants were recruited from 18 local governmental hospitals. Nurse staff meeting the following inclusion criteria were enrolled: (1) Employment at one of the 18 local governmental hospitals, (2) Ability to comprehend the questionnaire content, (3) Willingness to participate and providing informed consent, (4) No diagnosis of any mental illness, and (5) Not currently enrolled as student nurses.

For participant recruitment, a convenience sampling method was used, with individual information collected through a self-designed questionnaire administered via Wenjuanxing software, China’s largest online questionnaire platform. Prior to the survey, researchers underwent comprehensive training and provided a detailed explanation of the study’s purpose. The questionnaire link was distributed through the nursing departments of governmental hospitals, emphasizing privacy and independence in completing the questionnaire. Participants were assured of support and the right to withdraw from the study. The sampling calculation, conducted using the cross-sectional survey formula, resulted in 1774 qualified questionnaires out of 1965 nurse staff members invited, yielding a response rate of 90.3%.

### Measurement

#### Socio-demographic variables

The study’s socio-demographic characteristics encompassed age (in years), gender (women/men), ethnicity (Han/others), marital status (unmarried/married/divorced/others; categorized into unmarried/others), residence (rural/urban), education level (high school or lower/bachelor’s degree or above), only child status (yes/no), monthly income categories (3000 or lower/3001–5000/5001–7000/7000 or higher), among other variables.

#### Perceived cognitive deficits

The measurement of perceived cognitive deficits utilized the Perceived Deficit Questionnaire-5-Item (PDQ-5) [25], which comprises 5 items. Each item is rated on a scale from 0 (never) to 4 (always), with sum scores ranging from 0 to 20. A higher sum score reflects a greater level of perceived cognitive deficits. The PDQ-5 scale has demonstrated good reliability and validity in the Chinese population [26], with a Cronbach’s alpha of 0.85 reported in this study.

### Anxiety symptoms

Anxiety symptoms were assessed using the Generalized Anxiety Disorder-7 items (GAD-7) [27], consisting of 7 items. Each item is rated from 0 (never) to 3 (always), with sum scores ranging from 0 to 21. Higher sum scores indicate a higher level of anxiety symptoms. The GAD-7 scale has demonstrated good reliability and validity in the Chinese population [19], with a Cronbach’s alpha of 0.93 reported in this study.

### Depressive symptoms

Depressive symptoms were assessed using the Patient Health Questionnaire (PHQ-9) [28], which consists of 9 items. Each item is rated from 0 (never) to 3 (always), with total scores ranging from 0 to 27. A higher score indicates a higher level of depressive symptoms. The PHQ-9 scale has demonstrated good reliability and validity among the Chinese population [29], with a Cronbach’s alpha of 0.91 reported in this study.

#### Resilience

Resilience was assessed using the Connor-Davidson Resilience Scale-10 items (CD-RISC10) [30]. This scale employs a 5-point rating system from 0 (never) to 4 (always), resulting in a total score range of 0–40, where higher scores indicate a higher level of resilience. The CD-RISC10 scale has been reported to possess good reliability and validity within the Chinese population [31, 32], with a Cronbach’s Alpha of 0.95 in the present sample.

#### Childhood trauma

The Childhood Trauma Questionnaire-Short Form (CTQ-SF) [33] was utilized to assess childhood trauma. This self-report questionnaire evaluates traumatic experiences before the age of 16, encompassing various forms of abuse (physical, emotional, sexual) and neglect (physical, emotional). The CTQ-SF comprises 28 items, including 25 clinical items and 3 validity items. Responses are recorded on a 5-point Likert scale, ranging from 1 (never) to 5 (always), with the total score spanning from 25 to 125, where higher scores indicate a higher frequency of maltreatment. The instrument has been used in the Chinese population and demonstrated good reliability [34, 35], with a Cronbach’s Alpha of 0.73 reported in the present study.

### Social support

The level of social support was measured using the Multidimensional Scale of Perceived Social Support (MSPSS) [36], which comprises 12 items assessing social support from family (4 items), friends (4 items), and others (4 items). Each item is rated on a seven-point Likert scale, ranging from 1 (representing “strongly disagree”) to 7 (representing “strongly agree”). The total score of all items was computed for analysis purposes, with higher scores indicating a higher level of social support. The MSPSS has demonstrated good reliability and validity among the Chinese population [37, 38], with a Cronbach’s alpha of 0.96 reported in this study.

### Loneliness

The loneliness of participants was assessed using the Three-Item Loneliness Scale [39], which utilizes response categories coded as 1 (hardly ever), 2 (some of the time), and 3 (often). The total score ranges from 3 to 9, with higher scores indicating higher levels of perceived loneliness. This scale has been reported to possess good reliability and validity among the Chinese population [40, 41], with a Cronbach’s Alpha of 0.83 reported in this study.

### Sleep quality

The sleep quality throughout the past seven-day period was measured using a new single-item sleep quality scale [42], where respondents mark an integer score from 0 (terrible) to 10 (excellent) in the survey. A higher score indicates a higher level of sleep quality. This scale has been applied both domestically and internationally, with well-established reliability and validity.

### Suicidal ideation and non-suicidal self-injury

Suicidal ideation was assessed using a single-item question: “During the past 12 months, did you consider attempting suicide?“. Non-suicidal self-injury was measured by another single-item question: “During the past 12 months, did you engage in non-suicidal self-injury?“. Participants were asked to evaluate their experiences using a scale that ranged from “none” (coded as 0) to “always” (coded as 4). For analysis purposes, a score of 0 indicated no suicidal ideation or non-suicidal self-injury, while scores higher than 0 indicated the presence of suicidal ideation or non-suicidal self-injury. These two questions have been widely utilized in research conducted among the Chinese population.

### Statistical analysis

In the study, socio-demographic characteristics and psychological information were represented as Mean ± SD for continuous variables and N(%) for categorical variables. To compare categorical variables, the Chi-square test was conducted, while for comparison of continuous variables, One-way ANOVA was executed. The Z-scores of latent variables were used to indicate different sum scores, with a mean of 0 and a standard deviation of 1.

### Latent profile analysis (LPA)

Latent Profile Analysis (LPA) is a statistical method that employs a person-centered approach to identify potential groups from measurable continuous variables. In this study, perceived cognitive deficits, anxiety symptoms, depressive symptoms, resilience, childhood trauma, social support, loneliness, and high sleep quality were integrated into the LPA to characterize individuals based on the pattern of their complex identity attributes, thus creating distinct latent classes.

To determine the number of underlying classifications, a combination of model fit indexes should be considered, such as Loglikelihood, Akaike information criterion (AIC), Bayesian information criterion (BIC), Adjusted Bayesian information criterion (adj. BIC), Entropy, and Lo-Mendel-Rubin adjusted bootstrap likelihood ratio test (Adj. LRT). Lower values of AIC, BIC, and adj. BIC indicate better model fit. Entropy, which ranges from 0 to 1, measures classification accuracy; a value closer to 1 indicates higher accuracy, with values over 0.8 reflecting a classification accuracy of 90%. When the value of Adj. LRT is less than 0.05, it suggests that the k number of underlying classifications is better than the k-1 number of underlying classifications.

To enhance the generalizability of LPA results, a minimum threshold of 5% was set for the sample size ratio of each class [3].

### Logistic regression model

In the study, a binary logistic model was utilized to investigate the associations between latent classes and both suicidal ideation and non-suicidal self-injury. In these logistic regression models, suicidal ideation and non-suicidal self-injury were the dependent variables, while the latent pattern was the independent variable, controlling for socio-demographic characteristics. All regression analyses were conducted using SPSS 26.0, and LPA was performed using R software. A two-sided p-value of less than 0.05 was considered statistically significant in this study.

### Result

#### Latent profile analysis

**Fig. 1:**
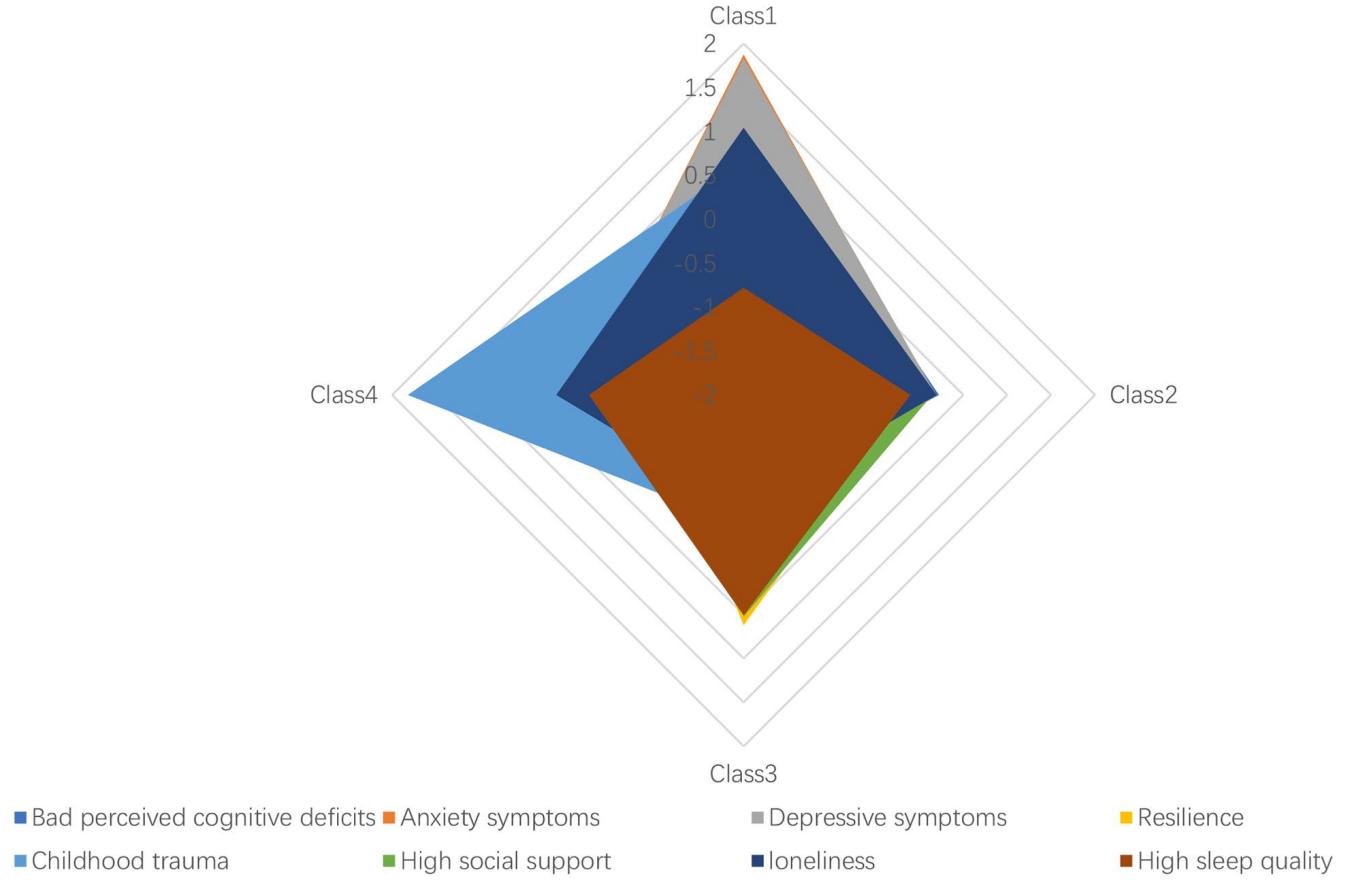
Latent classes by categorical Z-score mean

The LPA analysis, as detailed in Table 1, revealed a notable decline in the AIC index, BIC index, and Log-likelihood index as model number increased. The LRT test indicated that the 5-class model exhibited superior fit compared to the 4-class model, which in turn outperformed the 3-class model, and the 3-class model showed better fit than the 2-class model. Notably, the 6-class model did not significantly improve upon the 5-class model (*p* > 0.05). Considering the minimum sample size (N _least_, 5% of the total population) and other fit indicators, the 4-class model was deemed optimal for classification in this study. Additionally, Z-scores of 8 observed variables were utilized to compare their relative values, depicted in Fig. 1. Furthermore, socio-demographic characteristics and psychological information for the 4 classes and all participants were presented in Table 2, where the chi-square test results suggested potential differences in sex and ethnicity across the four classes.

**Table 1:** Latent class fit indices

Model	Log-likelihood	Entropy	AIC	BIC	Adj. BIC	Adj. LRT *P*-value		*N*_least_
2 class model	-41857.89	0.799	83765.78	83902.80	83823.37	<0.001	665 (37.5%)	
3 class model	-41245.24	0.852	82558.47	82744.83	82636.81	< 0.001		194 (10.9%)
**4 class model**	**-40860.04**	**0.857**	**81806.08**	**82041.77**	**81905.16**	**0.001**		**159 (9.0%)**
5 class model	-40601.19	0.876	81306.39	81591.40	81426.20	< 0.001		71 (4.0%)
6 class model	-40373.91	0.874	80869.82	81204.16	81010.37	0.243		70 (3.9%)

Note: AIC = Akaike information criteria; BIC = Bayesian information criteria; LRT = Lo-Mendell-Rubin test

**Table 2:** Socio-demographic characteristics of the sample (*N* = 1774)

	Full sample		Class 1		Class 2		Class 3		Class 4	
	Mean/*N*	SD/%	Mean/*N*	SD/%	Mean/*N*	SD/%	Mean/*N*	SD/%	Mean/*N*	SD/%
**Latent variables**										
Perceived cognitive deficits	7.12	4.27	12.60	3.60	8.07	3.34	3.59	2.58	7.34	4.15
Anxiety symptoms	6.29	4.32	14.39	3.53	7.00	2.24	2.28	2.08	6.16	2.98
Depressive symptoms	7.42	5.13	16.81	4.37	8.39	2.75	2.53	2.25	7.35	3.33
Resilience	21.85	8.28	17.69	6.29	20.97	6.03	26.99	9.09	13.93	7.06
Childhood trauma	36.86	10.73	43.84	12.71	35.09	7.22	31.47	6.53	56.42	9.48
Social support	62.60	14.02	54.10	14.41	64.65	9.22	69.94	8.98	36.65	14.69
loneliness	2.26	1.55	3.88	1.44	2.56	1.26	1.15	1.16	2.47	1.66
High sleep quality	6.33	2.44	4.44	2.38	6.09	2.28	7.58	2.07	5.75	2.34
**Age** (T = 97.03, *P* = 0.678)	14.99	14.66	32.27	6.89	31.76	7.67	32.19	8.84	32.28	7.94
**Sex**	**χ**^2^ **= 25.20*P* < 0.001**									
Women	1666	93.9	192	95.5	822	95.6	516	93.1	136	85.5
Men	108	6.1	9	4.5	38	4.4	38	6.9	23	14.5
**Ethnic**	**χ**^2^ **= 10.67*P* = 0.014**									
Han	1276	71.9	146	72.6	606	70.5	422	76.2	102	64.2
Others	498	28.1	55	27.4	254	29.5	132	23.8	57	35.8
**Marital status**	χ^2^ = 12.20 *P* = 0.058									
married	517	29.1	46	22.9	258	30.0	178	32.1	35	22.0
Unmarried	1200	67.6	146	72.6	579	67.3	358	64.6	117	73.6
Divorce/others	57	3.2	9	4.5	23	2.7	18	3.2	7	4.4
**Residence**	χ^2^ = 2.608 *P* = 0.456									
Rural	1071	60.4	115	57.2	525	61.0	328	59.2	103	64.8
Urban	703	39.6	86	42.8	335	39.0	226	40.8	56	35.2
**Education level**	χ^2^ = 2.44 *P* = 0.486									
High school or lower	614	34.6	60	29.9	299	34.8	198	35.7	57	35.8
Bachelor degree or above	1160	65.4	141	70.1	561	65.2	356	64.3	102	64.2
**Only children**	χ^2^ = 0.97 *P* = 0.808									
Yes	283	16.0	36	17.9	136	15.8	84	15.2	27	17.0
No	1491	84.0	165	82.1	724	84.2	470	84.8	132	83.0
**Income (monthly)**	χ^2^ = 9.42 *P* = 0.399									
3000 or lower	498	28.1	58	28.9	237	27.6	154	27.8	49	30.8
3001–5000	782	44.1	94	46.8	382	44.4	233	42.1	73	45.9
5001–7000	325	18.3	33	16.4	164	19.1	100	18.1	28	17.6
7000 or higher	169	9.5	16	8.0	77	9.0	67	12.1	9	5.7

Class 1, termed “emotional problem group” (EP group), encompassed 201 individuals (11.33%), with anxiety symptoms exhibiting the highest score among all psychosocial factors, followed by depressive symptoms, perceived cognitive deficits, and loneliness. Class 2, identified as the “lower emotional problem group” (LEP group), comprised 860 individuals (48.48%). Although this class demonstrated the highest scores across all psychosocial factors, the score for perceived cognitive deficits was only 0.17 times that of Class 1’s score. Class 3, known as the “resilience and social support group” (RS group), included 554 individuals (31.23%), with resilience achieving the highest score among all psychosocial factors, closely followed by high social support. Lastly, Class 4, designated as the “childhood trauma group” (CT group), consisted of 159 individuals (8.96%), with childhood trauma scoring highest among all psychosocial factors.

### Logistic regression analysis

The logistic regression analysis in Table 3 focuses on suicidal ideation and non-suicidal self-injury. Both models, one for suicidal ideation (χ² = 2.89, df = 8, *P* = 0.941) and the other for non-suicidal self-injury (χ² = 3.46, df = 8, *P* = 0.902), exhibit good fit according to the Hosmer and Lemeshow test.

**Table 3:** Logistic regression analysis of suicidal ideation and suicidal attempt by latent classes

Variables	Suicidal ideation		Non-suicidal self-injury	
	OR	95%CI	OR	95%CI
**Latent classes (class 2 as ref)**				
Class 1	**6.59**	**4.42–9.81**	**5.13**	**3.38–7.78**
Class 3	**0.21**	**0.11–0.42**	**0.15**	**0.07–0.36**
Class 4	**2.12**	**1.25–3.62**	**2.13**	**1.25–3.65**
**Age**	0.98	0.95–1.02	1.003	0.97–1.04
**Sex**				
Women	ref			
Men	0.60	0.26–1.40	1.35	0.64–2.82
**Ethnic**				
Han	ref			
Others	1.24	0.85–1.80	0.97	0.65–1.45
**Marital status**				
Married	ref			
Unmarried	**0.58**	**0.37–0.91**	1.20	0.73–1.96
Divorce/others	**2.34**	**1.02–5.35**	**2.58**	**1.001–6.65**
**Residence**				
Rural	ref			
Urban	1.17	0.76–1.81	1.06	0.68–1.67
**Education level**				
High school or lower	ref			
Bachelor degree or above	0.90	0.61–1.32	0.80	0.53–1.20
**Only children**				
No	ref			
Yes	0.85	0.51–1.42	0.82	0.47–1.41
**Income (monthly)**				
3000 or lower	ref			
3001–5000	1.28	0.81–2.02	1.12	0.70–1.80
5001–7000	1.64	0.88–3.06	1.05	0.54–2.03
7000 or higher	2.26	0.97–5.27	0.88	0.35–2.25
Nagelkerke R^2^	0.204		0.171	
Model fit (Hosmer and lemeshow)	χ^2^ = 2.89, DF = 8, *P* = 0.941		χ^2^ = 3.46, DF = 8, *P* = 0.902	

Compared to individuals in class 2, those classified in class 1 faced a sixfold higher risk of suicidal ideation (OR = 6.59, 95% CI = 4.42–9.81) and a fivefold higher risk of non-suicidal self-injury (OR = 5.13, 95% CI = 3.38–7.78). Similarly, individuals in class 4 had double the risk of suicidal ideation (OR = 2.13, 95% CI = 1.25–3.62) and non-suicidal self-injury (OR = 2.13, 95% CI = 1.25–3.65) compared to those in class 2. Conversely, individuals in class 3 were more likely to have lower risks of suicidal ideation (OR = 0.21, 95% CI = 0.11–0.42) and non-suicidal self-injury (OR = 0.15, 95% CI = 0.07–0.36) than individuals in class 2.

Furthermore, individuals with a marital status of divorce or others had a higher risk of suicidal ideation (OR = 2.34, 95% CI = 1.02–5.35) and non-suicidal self-injury (OR = 2.58, 95% CI = 1.01–6.65) compared to married individuals. On the contrary, individuals with an unmarried marital status had a lower risk of suicidal ideation (OR = 0.58, 95% CI = 0.37–0.91).

## Discussion

This study employed Latent Profile Analysis (LPA) to investigate distinct levels of suicidal ideation and non-suicidal self-injury among different subgroups of nurses. The analysis identified four latent patterns based on eight observable psychosocial characteristics. Individuals with a marital status of divorce or others showed a higher risk of suicidal ideation (OR = 2.34, 95% CI = 1.02–5.35) and non-suicidal self-injury (OR = 2.58, 95% CI = 1.01–6.65) compared to married individuals, while unmarried individuals had a lower risk of suicidal ideation (OR = 0.58, 95% CI = 0.37–0.91). Regression analysis also revealed a gradual increase in risk as profiles shifted from class 3 to class 2, then to class 4, and ultimately to class 1. These findings highlight the importance of considering socio-demographic factors in understanding suicidal behaviors among nurses. Additionally, the identified subgroups, including the emotional problem group, lower emotional problem group, resilience and social support group, and childhood trauma group, underscore the diverse psychosocial characteristics present among nurses and their potential reciprocal impacts.

The analysis identified four latent patterns among nurses based on eight observable psychosocial characteristics, delineating the “emotional problem group” (EP group) with elevated anxiety symptoms, depressive symptoms, bad perceived cognitive scores, and loneliness, coupled with lower sleep quality. The “lower emotional problem group” (LEP group) also exhibited high scores in these areas, albeit notably lower than those of the EP group. Furthermore, the “resilience and social support group” (LS group) demonstrated positive resilience, high social support, and favorable sleep quality, while other psychological factors reflected contrasting outcomes. Lastly, the “childhood trauma group” (CT group) displayed the highest childhood trauma scores and relatively diminished social support. These findings align with prior research [43], emphasizing the co-existence of diverse psychosocial characteristics within individuals and their potential reciprocal impacts.

In our study, we observed that individuals with a marital status of divorce or other statuses exhibited a significantly higher risk of suicidal ideation and non-suicidal self-injury compared to their married counterparts. Consistent with previous studies [44, 45], this finding highlights the substantial influence of marital status on an individual’s mental health and well-being. Additionally, Yating Wei’s study indicated that single individuals are more likely to experience mental health issues [46]. Conversely, in our study, we found that unmarried individuals demonstrated a lower risk of suicidal ideation. This difference may be attributed to the characteristics of our study population—nurses who typically have stable jobs and relatively higher incomes, suggesting a preference for remaining single rather than being influenced by mental health problems that often accompany singlehood as seen in Yating Wei’s sample derived from a retrospective study based on the psychological assistance hotline in Hangzhou. These results highlight the complex interplay between marital status and mental health outcomes, emphasizing the need for a more nuanced understanding of the role of social support and interpersonal relationships in suicide prevention efforts.

It is important to highlight that in our study, class 1, labeled as the “EP group,” emerged as the highest-risk group for both suicidal ideation and non-suicidal self-injury. This finding aligns with a previous study [47] conducted on sexual minority adolescents using LPA, which revealed that “adolescents with mood problems” represented the highest risk group for suicidal ideation, characterized by elevated anxiety, depressive symptoms, and poor sleep quality. Prior research has consistently demonstrated positive correlations between mental health issues such as anxiety and depressive symptoms and the prevalence of suicidal ideation and non-suicidal self-injury [11, 12, 16, 19, 22, 23]. Both anxiety and depressive symptoms have been associated with changes in an individual’s biological and psychosocial well-being, subsequently impacting sleep patterns [48]. Consequently, it becomes plausible to understand why the EP group exhibited heightened anxiety and depressive symptoms alongside lower sleep quality. Furthermore, loneliness has been shown to positively predict anxiety and depressive symptoms [49]. Not only does loneliness negatively impact cognitive functioning, but it also has the potential to exacerbate anxiety [50]. The interplay of these psychological variables within class 1 may contribute to a higher risk of suicidal ideation and non-suicidal self-injury compared to each variable in isolation.

In a comparison between the “LEP group” (class 2) and “LS group“(class 3), our analysis revealed that individuals in the “LS group” demonstrated a lower risk of both suicidal ideation and non-suicidal self-injury. This finding aligns with existing literature emphasizing the pivotal role of social support and resilience as protective factors against suicidality [12, 13, 21]. Resilience, defined as an individual’s capacity to adapt to adverse events, plays a crucial role in mitigating anxiety and depression [51, 52]. High levels of resilience are associated with reduced levels of anxiety and depression [53, 54]. On the other hand, social support provides valuable psychological and material resources from one’s social network, serving as a coping mechanism to alleviate psychological distress [55]. These insights deepen our understanding of the complex interplay between psychosocial factors and suicidal behaviors among nurses, underscoring the importance of promoting resilience and fostering social support as key components of intervention and prevention strategies within healthcare environments.

Conversely, individuals classified in the “CT group” (class 4) exhibited a higher risk of suicidal ideation and non-suicidal self-injury compared to those in the “LEP group” (class 2). Childhood trauma has been consistently associated with these adverse outcomes. Existing research underscores the critical role of social support and resilience in mitigating suicidality [12, 13, 21]. When individuals are confronted with multiple risk factors and lack protective factors simultaneously, the likelihood of experiencing negative consequences significantly escalates. Interventions aimed at increasing protective factors or reducing exposure to risk factors can effectively diminish the overall risk of these issues. This highlights the importance of addressing childhood trauma, enhancing social support, and fostering resilience as key components in comprehensive strategies to prevent and manage suicidal behaviors among nurses and other populations in healthcare settings.

### Limitations of the study

The study is subject to several methodological limitations. Firstly, the use of a cross-sectional design restricts the capacity to establish causal relationships between variables. Second, the application of Latent Profile Analysis (LPA) to assess the impact of psychosocial characteristics on suicidal ideation and non-suicidal self-injury among nurse staff was based on a selection of specific variables, potentially overlooking other relevant psychosocial factors not accounted for in the model. Third, the recruitment of participants solely from the Dehong districts in Yunnan province using convenience sampling may limit the generalizability of the findings to nursing staff across China. Finally, the reliance on self-reported data introduces the potential for self-reporting bias, as participants may selectively disclose or conceal certain information. These limitations underscore the need for cautious interpretation of the results and highlight areas for further research to address these constraints and enhance the robustness of future findings.

### Strengths

This study’s strengths are evident in its methodological sophistication, characterized by the use of Latent Profile Analysis (LPA) to identify latent subgroups based on observable traits, leading to a more detailed understanding of the studied population. Additionally, the comprehensive assessment of various psychosocial factors like anxiety, depression, resilience, and childhood trauma provides profound insights into the complex interplay influencing suicidal ideation and non-suicidal self-injury. Moreover, the study’s ability to pinpoint specific risk groups at higher susceptibility to such behaviors highlights its capacity to inform targeted prevention and intervention strategies, emphasizing the importance of tailored support frameworks for at-risk individuals in professions with elevated stress levels.

## Conclusion

Based on eight key psychosocial factors including Perceived cognitive deficits, anxiety symptoms, depressive symptoms, resilience, social support, childhood trauma, loneliness, and sleep quality, nurse staff were categorized into four latent pattern classes. In comparison to “class 2,” both “class 1” and “class 4” demonstrated a higher likelihood of experiencing suicidal ideation and non-suicidal self-injury, while “class 3” displayed a reduced risk of these outcomes. When designing interventions targeted at preventing suicide among nurse staff, a focus on psychosocial characteristics is crucial, particularly directing attention towards individuals in “class 1” and “class 4” with elevated risks. Apart from mental health guidance, enhancing social support and resilience stands out as essential for nurse staff exhibiting higher levels of suicidal ideation and non-suicidal self-injury. Future research efforts should prioritize investigating interventions tailored towards the needs of individuals in “class 1” and “class 4” for more effective targeted strategies.

## Data Availability

The datasets generated during and/or analysed during the current study are available from the corresponding author on reasonable request.

## Abbreviations

PDQ-5: Perceived Deficit Questionnaire-5-item, GAD-7:The generalized anxiety disorder-7
CDRISC-10: The Connor Davidson Resilience Scale – 10 item
PHQ-9: The 9-item Patient Health Questionnaire
MSPSS: Multidimensional Scale of Perceived Social Support
CTQ-SF: Childhood Trauma Questionnaire-Short Form
